# Adenosine Signaling in Mast Cells and Allergic Diseases

**DOI:** 10.3390/ijms22105203

**Published:** 2021-05-14

**Authors:** Lucia Garcia-Garcia, Laia Olle, Margarita Martin, Jordi Roca-Ferrer, Rosa Muñoz-Cano

**Affiliations:** 1 Clinical and Experimental Respiratory Immunoallergy (IRCE), Institut d’Investigacions Biomèdiques August Pi i Sunyer (IDIBAPS), 08036 Barcelona, Spain; luciagg@clinic.cat (L.G.-G.); lolle@clinic.cat (L.O.); martin_andorra@ub.edu (M.M.); rocaferrer@gmail.com (J.R.-F.); 2 Biochemistry and Molecular Biology Unit, Biomedicine Department, Faculty of Medicine, University of Barcelona, 08036 Barcelona, Spain; 3 ARADyAL, Instituto de Salud Carlos III, 28220 Madrid, Spain; 4 Allergy Section, Hospital Clinic, Universitat de Barcelona, 08036 Barcelona, Spain

**Keywords:** adenosine, adenosine receptors, G protein, mast cells, allergic diseases, asthma

## Abstract

Adenosine is a nucleoside involved in the pathogenesis of allergic diseases. Its effects are mediated through its binding to G protein-coupled receptors: A1, A2a, A2b and A3. The receptors differ in the type of G protein they recruit, in the effect on adenylyl cyclase (AC) activity and the downstream signaling pathway triggered. Adenosine can produce both an enhancement and an inhibition of mast cell degranulation, indicating that adenosine effects on these receptors is controversial and remains to be clarified. Depending on the study model, A1, A2b, and A3 receptors have shown anti- or pro-inflammatory activity. However, most studies reported an anti-inflammatory activity of A2a receptor. The precise knowledge of the adenosine mechanism of action may allow to develop more efficient therapies for allergic diseases by using selective agonist and antagonist against specific receptor subtypes.

## 1. Introduction

**Figure 1:**
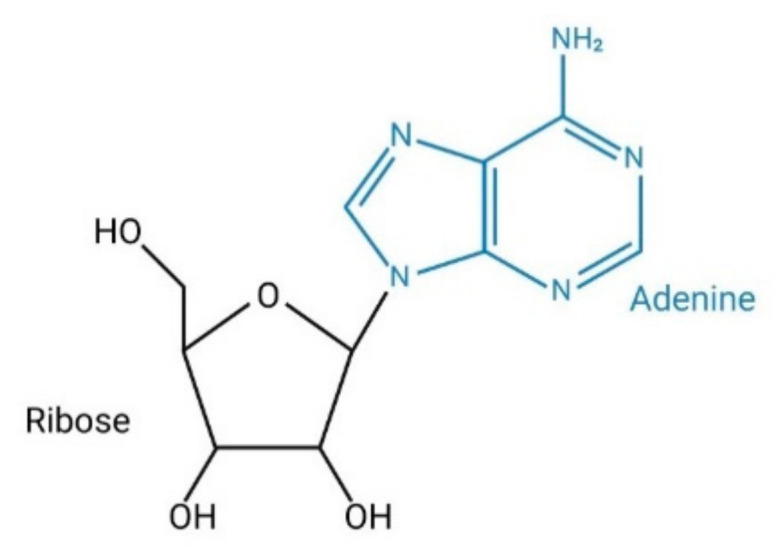
Structure of adenosine.

Adenosine is an endogenous purine nucleoside consisting of the union, through a glycosidic linkage, of an adenine with a sugar ribose (Figure 1) [1]. It is an intermediary metabolite that plays a vital role in the synthesis of nucleic acids and forms adenosine triphosphate (ATP), the main component of the cellular energy, adenosine diphosphate (ADP) and adenosine monophosphate (AMP). Adenosine is involved in various physiological and pathological functions including the modulation of inflammatory processes such as allergic diseases [2].

### 1.1. Synthesis and Degradation of Adenosine

Under physiological conditions, a low adenosine concentration (from 10 to 200 nM) is present in the extracellular space. However, under conditions of metabolic stress and in certain pathological situations, there is a significant elevation of adenosine concentration, reaching levels that can be as high as 100 µM [3,4].

**Figure 2:**
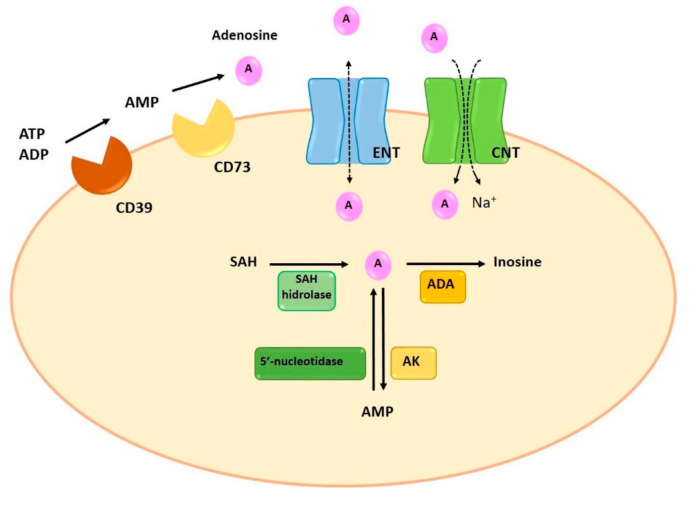
Synthesis, degradation and transport of adenosine. Intracellularly, adenosine is generated by dephosphorylation from AMP, by 5′-nucleotidase, or by hydrolysis of SAH, by SAH hydrolase. Extracellular adenosine generation is the result of the enzymatic activity of CD39 and CD73. ENT and CNT transporters allow the reuptake of adenosine. Finally, the adenosine is metabolized intracellularly by two enzymes, ADA and AK, which will produce inosine and AMP. ADA: adenosine deaminase; AK: adenosine kinase; AMP: adenosine monophosphate; CNT: concentrative nucleoside transporters; ENT: equilibrative nucleoside transporters; SAH: S-adenosyl-homocysteine.

Adenosine can be synthesized at intracellular and extracellular levels. In physiologic conditions adenosine is generated in the intracellular space while, under metabolically unfavorable conditions, extracellular generation is carried out. Intracellular synthesis is dependent on the dephosphorylation of AMP by 5′-nucleotidase or the hydrolysis of S-adenosyl-homocysteine (SAH) by SAH hydrolase [5] (Figure 2). Extracellular generation is carried out through a mechanism that involves the dephosphorylation of ATP, ADP, and AMP. The extracellular synthesis of adenosine is regulated by the sequential action of two enzymes on the cell surface: ectonucleoside triphosphate diphosphohydrolase-1 (CD39) and 5′-ectonucleotidase (CD73). CD39 presents ATPase and ADPase activity and therefore can dephosphorylate both ATP and ADP, forming AMP. The last step for the generation of adenosine is carried out by CD73 through the dephosphorylation of AMP [6] (Figure 2). Since the adenosine precursors (ATP, ADP and AMP) are released by cells in situations of stress, inflammation and hypoxia, pathological situations will result in a large increase in the concentration of adenosine [7,8]. Similarly, the enzymes involved in their extracellular synthesis (CD39 and CD73) are overexpressed in some pathological situations, such as hypoxia and chronic obstructive pulmonary disease, resulting in increases of adenosine synthesis [9,10,11]. Finally, an attenuation of allergic airway inflammation has been observed in a CD39^-/-^ mouse model [12].

Adenosine degradation is also involved in the final extracellular concentration of adenosine. This process can be performed in both, the extracellular and the intracellular space. Extracellularly, adenosine can be metabolized by the enzyme adenosine deaminase (ADA), which causes the deamination of adenosine into inosine. ADA can deaminate the adenosine when is anchored to the plasma membrane through its binds to membrane proteins such as CD26 [13]. However, the pathway that involves the activity of extracellular ADA is not the main way of adenosine degradation [14].

Normally, adenosine degradation takes place in the intracellular space. In fact, the adenosine present in the intracellular space can be directly metabolized while extracellular adenosine can suffer extracellular deamination by ADA or can be transferred to the intracellular compartment by specific transporters. The adenosine reuptaked to the intracellular compartment is rapidly metabolized through two metabolic pathways: ADA and adenosine kinase (AK). Similar to the extracellular metabolism, the enzymatic activity of ADA deaminates the adenosine to inosine. The second pathway involves the activity of AK, which phosphorylates adenosine and converts it into AMP [5] (Figure 2).

ADA activity has been related with some allergic diseases. A severe lung inflammation with eosinophilic airway infiltration and mast cell degranulation, features found in asthma patients, have been observed in an ADA-deficient mice model. Treatment with exogenous ADA, that reduced adenosine concentrations, resulted in the reversal of the inflammation [15]. Interestingly, an increase in inosine levels, which has been shown to increase RBL-2H3 rat basophil line degranulation through A3 AR activation [16], was also observed in a mouse model of allergic asthma, suggesting also the role of inosine in allergy inflammation [17].

### 1.2. Adenosine Transporters

Extracellularly, adenosine concentration is regulated by reuptake mechanisms through the action of two specific transporters: concentrative nucleoside transporters (CNTs) and equilibrative nucleoside transporters (ENTs) [18]. CNTs are active Na^+^-dependent transporters, mediate cell influx of nucleosides in the presence of an inwardly directed sodium gradient, against its concentration gradient. ENTs are passive bidirectional transporters which transport adenosine across the plasma membrane based on concentration gradients [19,20]. Despite both transporters participate in the regulation of adenosine extracellular concentration, it has been reported that ENTs are the most relevant transporters of adenosine, since they determine both the release and the reuptake of adenosine [9] (Figure 2).

## 2. Adenosine Receptors

Adenosine mediates its effects through activating G protein- coupled receptors (GPCRs). There are four types of adenosine receptors (ARs): A1, A2a, A2b and A3. All of them have a core domain which crosses the plasma membrane seven times, a 20–27 amino acids long helix and linked by three intracellular and three extracellular loops. The extracellular N- terminus presents one or more glycosylation sites, while the intracellular C-terminus has phosphorylation and palmitoylation sites, which have an important role for receptor internalization and desensitization [21]. ARs differ in the number of amino acids they present: A1 consists of 326 amino acids; A2a is the largest with 412 amino acids; A2b consists of 332 amino acids; and A3 presents 318 amino acids [22].

**Figure 3:**
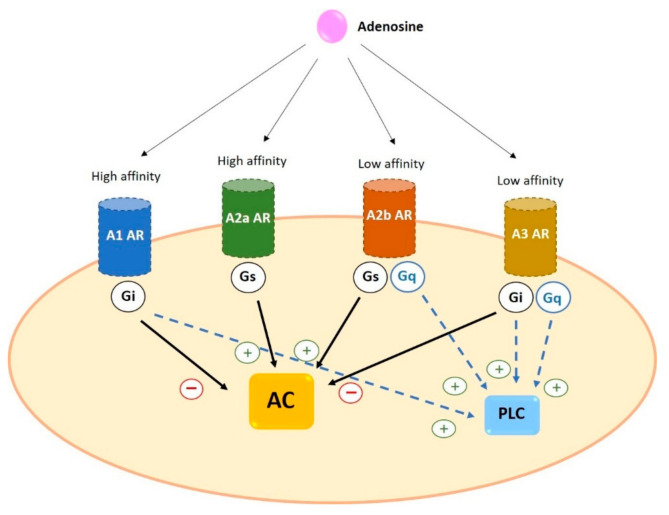
General characteristics of adenosine receptors. A1 and A2a ARs have a higher affinity for adenosine than A2b and A3 ARs. All four receptors trigger AC activation (via Gs) or inhibition (via Gi). In addition, some ARs can activate PLC coupling to Gq or through Gi. Black arrows indicate the effect on AC; blue dotted arrows indicate the effect on PLC. AC: adenylyl cyclase AR: adenosine receptors PLC: phospholipase C.

Adenosine receptors have different affinity for adenosine: A1 and A2a possess high affinity while A2b and A3 show relatively lower affinity [20]. In addition, as a general characteristic, receptors differ in the type of G protein they recruit, their effect on adenylyl cyclase (AC) activity and the downstream signaling pathway triggered. A1 and A3 are coupled to G_i_ and inhibit the activity of AC while A2a and A2b preferentially couple to G_s_ and increase cAMP levels by stimulation of AC [5,19] (Figure 3).

### 2.1. Expression of Adenosine Receptors in Human Cells

Adenosine receptors are widely distributed in human cells. These receptors play a role in diverse biological functions and show a broad spectrum of action [23]. The A1 receptor is the most conserved and it is expressed with the highest levels in the central nervous system (CNS), mainly in the neocortex, cerebellum, hippocampus, autonomic nerve terminals, spinal cord, and glial cells [24]. A1 is also found with high abundance in heart, kidney, adipose tissue, and pancreas [25]. Furthermore, and related to inflammatory airway diseases, it has been demonstrated that A1 is expressed in alveolar epithelial cells, airway smooth muscle cells, and several immune cells such as neutrophils, macrophages and monocytes, where A1 enhances proinflammatory effects [7].

A2a is found in heart, lung, liver, nervous and immune system. High A2a levels are present in the striatum of the brain and the olfactory tubercle, lymphocytes, neutrophils, macrophages, monocytes, and dendritic cells [21].

The highest levels of A2b are essentially found in the periphery, in the urinary blander, bowel, lung, vas deferens, and different cell types including, smooth muscle, alveolar epithelial, chromaffin and taste cells, as well as immune cells such as mast cells, neutrophils, dendritic, neutrophils and lymphocytes [3]. At the central level, the A2b is expressed in astrocytes, microglia and neurons [7].

A3 is expressed at the highest levels in lung, liver and immune cells. Lower levels have been reported in the heart and brain [5,26].

#### Expression of Adenosine Receptors in Human Basophils and Mast Cells

Mast cells and basophils play a pivotal role in the pathophysiology of allergic diseases through the secretion of pro-inflammatory mediators. Both types of cells are usually activated by the cross-linking of the allergen with its specific immunoglobulin E (IgE) and its binding to the high affinity IgE receptor (FcεRI) [27].

The ARs profile expression seems to be different when comparing mast cells with basophils. Moreover, different ARs expression has been reported depending on the source of the mast cells. For instance, it has been reported that human skin mast cells express all four receptors while human peripheral basophils express A2a, A2b and A3, but not A1 [28]. However, studies carried out in the human mast cell line LAD2 [29] and HMC-1 cells [30] demonstrated that both cell lines express A2a, A2b and A3, but not A1. In addition to the different expression profile, it has been reported differences in the level of expression. Gomez et al. demonstrated that A3 messenger RNA (mRNA) expression in human lung mast cells was threefold higher than in skin mast cells, difference that may account for their disparity in the response to adenosine [31]. Muñoz-Cano et al. reported a higher expression of A3 in food anaphylaxis patients compared to health individuals, although the study was performed in whole blood samples where basophils, but not mast cells, are represented [32]. Similarly, using whole blood samples, polymorphisms of ADORA3 have been identified in patients with aspirin-induced urticaria [33], of ADORA1 and ADORA2A in aspirin-induced asthma [34], both in Korean population; ADA polymorphism have been observed in asthma patients from a Chinese Han population [35]. However, as far as we know, it has not been reported differences between healthy subjects and allergic patients in the adenosine receptor profile of basophils or mast cells [36].

### 2.2. Signal Transduction of Adenosine Receptors in Human Cells

#### 2.2.1. A1 

A1 is coupled to G_i/o_ protein. Its activation leads to inhibition of the AC, causing the reduction of intracellular production of cAMP. A1 also involves phospholipase C (PLC) activation and results in an increase in inositol 1,4,5-triphosphate (IP_3_) and intracellular Ca^2+^ levels. High levels of Ca^2+^ stimulate protein kinase C (PKC) and other calcium-binding proteins [37]. In cardiac muscle and neurons, A1 can stimulate potassium channels and inhibit Q-, P- and N-type calcium channels. A1 activation is related to the phosphorylation of mitogen- activated protein kinases p38, ERK1/2, and JNK1/2 [7].

#### 2.2.2. A2a

A2a binds to G_s_ protein and increases AC activity and cAMP production. The cAMP-dependent protein kinase A (PKA) is the main pathway, causing the phosphorylation and activation of several proteins such as the transcription factor cAMP- response element binding (CREB). Moreover, it has been reported that A2a is involved in the regulation of MAPK signaling [38].

#### 2.2.3. A2b

A2b is coupled to G_s/q_ proteins. The main signaling pathway, through binding to G_s_, involves the AC activation that leads to an increase in cAMP levels. As a result there is an activation of PKA by phosphorylation and others cAMP- dependent effectors such as Epac [3]. However, A2b can couple to G_q_ protein which mediates the activation of PLC [39] and resulting in Ca^2+^ mobilization. In addition, this type acts as stimulator of MAPK through phosphorylation of p38, ERK 1/2 and JNK 1/2 [7].

#### 2.2.4. A3 

A3 can couple to G_i_ protein to inhibit AC activity and decrease the level of cAMP. Moreover, A3 can also bind to G_q_ protein to stimulate PLC and increase intracellular concentration of Ca^2+^, action that can also be performed by Gβγ subunits. Furthermore, like other adenosine receptors, the A3 acts on MAPK and mediates the phosphorylation and activation of p38, ENK 1/2 and JNK 1/2 [40].

### 2.3. Desensitization of Adenosine Receptors

It has been reported that ARs can be desensitized due to the action of G protein-receptor kinases (GRKs) on the phosphorylation sites at the C-terminus of the GPCRs [41].

The activity of GRKs results in the internalization and desensitization when GPCRs are recruited from arrestins [41]. The A1, A2a, A2b and A3 have been shown to desensitize after stimulating with an agonist, but the rate of the desensitization depends on the AR type [42]: A3 desensitization is the fastest whereas A1 desensitization is the slowest [30,43,44]. Regulation of adenosine receptors is a complex process. Desensitization produces a reduction in the effects of adenosine receptors, which must be considered when designing possible agonists. For instance, the rapid desensitization of the A3 receptor suggests that an agonist could have the same effect as a specific antagonist [41].

## 3. Adenosine in Allergic Diseases

It has been suggested that adenosine is involved in the pathogenesis of allergic diseases such as asthma and urticaria. In fact, the studies focused in the analysis of the adenosine effect in patients suffering from asthma and urticaria suggested that adenosine play a role in these diseases. However, the results of the studies assessing the adenosine effects in mast cells and basophils are less conclusive.

In asthmatic patients, the inhalation of adenosine produces bronchoconstriction. However, this effect is not observed in healthy subjects [29]. In fact, it has been reported that the dysfunction of small airways plays a role in the dyspnea caused by adenosine in asthmatic patients [45]. Furthermore, high concentration of adenosine in bronchoalveolar lavage fluid and condensation of exhaled air have been reported in asthmatic patients [46]. In line with these findings, Mao et al. demonstrated that patients with urticaria have higher levels of plasma adenosine compared to healthy subjects [47].

Studies performed in mast cells seems to support the role of adenosine in these allergic diseases. In the human mast cell line LAD2, stimulation of the FcεRI pathway was enhanced by the incubation with low concentrations (10 nM–2 µM) of the adenosine analog NECA, which acts non-selectively on receptors. However, the application of selective agonists did not show a significant increase in degranulation, suggesting that interaction between different receptors may play a role in the stimulation of the FcεRI pathway [48]. In line with these findings, it has been demonstrated that low concentrations (<1 µM) of adenosine increased the histamine release induced by FcεRI in mast cells derived from human lung. However, high adenosine concentrations (1000 µM) produced an inhibition of the action of FcεRI in the same cells [49]. Supporting these findings, Matsuo et al. have reported that histamine release was inhibited in peripheral human basophils and human skin mast cells by adenosine at 1–1000 µM and 1–100 µM, respectively [28].

It seems that the different response to adenosine may be related with the origin of mast cells. Adenosine at low concentration (1 µM) produced a potentiation of the response induced by FcεRI in lung but not in skin mast cells. On the other hand, high concentrations of adenosine (1000 µM) produced an inhibition of β-hexoaminidase release both in lung and skin mast cells. In this study the expression of the receptors was quantified, demonstrating that lung mast cells had A3 overexpressed, compared with skin mast cells. This different pattern of expression could explain, at least in part, the differential response when cells were incubated with adenosine [31].

### 3.1. Role of Specific Receptor Stimulation in Allergic Diseases

#### 3.1.1. A1

Activation of this receptor induces both pro-inflammatory and anti-inflammatory effects in allergic diseases. It has been reported a low presence of A1 in the lung [19] a no expression in human lung mast cells [31]. However, several studies suggested that A1 is involved in airway inflammatory diseases pathogenesis. It has been demonstrated that A1 is upregulated in both bronchial epithelium and smooth muscle of asthmatic patients. Moreover, it has been demonstrated that its activation produced bronchoconstriction [46]. Supporting this finding, studies performed in animal models have reported that adenosine and allergen exposition induce bronchoconstriction [50]. On the contrary, in a model of ischemia-reperfusion lung, A1 activation showed an anti-inflammatory effect by reducing the neutrophil chemotaxis and edema [51]. Indeed, a reduction of microvascular permeability and polymorphonuclear cell trafficking in lipopolysaccharide (LPS)-damaged lung was also observed [19].

#### 3.1.2. A2a 

This receptor is characterized by its anti-inflammatory effects [19,52]. In A2a^-/-^ mouse models, there was an increase in the accumulation of pro-inflammatory cytokines in serum after treatment with an endotoxin compared to A2a ^+/+^ [53], indicating its anti-inflammatory effects. In rats, pre-treatment with a A2a selective agonist (CGS21680) before cardiovascular bypass has been shown to dampen lung injury and inflammation [54]. In line with these findings, Alfaro TM et al. have demonstrated that CGS21680 produces an attenuation of the inflammatory response of human alveolar macrophages. In fact, the pro-inflammatory stimulus induced the expression of A2a in these cells [55]. Supporting this anti-inflammatory role, it has also been reported that CGS21680 treatment during allergen sensitization or re-exposure resulted in a decrease in IFN-γ as well as the accumulation of neutrophils, lymphocytes, and eosinophils in bronchoalveolar lavage [56]. Moreover, A2a upregulation has been also reported in monocytes from bronchoalveolar lavage of asthmatic compared to healthy subjects [57]. This finding suggests inflammatory environment seems to cause the overexpression and activation of A2a, which could attenuate the pathological situation.

Human lung mast cells have Ca^2+^-activated K^+^ channel K_Ca_3.1, which are opened upon FcεRI activation, allowing calcium influx and degranulation [58]. A2a has been shown to inhibit the secretion of pro-inflammatory mediators induced by FcεRI pathway activation in mast cells, by means of closing K_Ca_3.1 channels [59]. Likewise, CSG21680 has shown to inhibit C3a-induced activation in LAD2 by decreasing intracellular calcium influx [60].

Current treatments for some inflammatory lung diseases such as asthma are mostly based on glucocorticoids, although some patients have shown a poor response to these treatments and new therapies with A2a specific agonist may be a promising option. However, treatments with the specific agonists GW-328267 or UK-432097 have shown low efficacy [38].

#### 3.1.3. A2b

The A2b receptor can bind to the Gs and Gq proteins, triggering both anti-inflammatory and pro-inflammatory effects [7]. The anti-inflammatory effects have been demonstrated in human and animal models and several cell types. In an A2b^-/-^ mouse model, an increase in FcεRI-induced mast cell activation was observed, suggesting the inhibitory role of this receptor [61]. In neutrophils, the activation of A2b inhibited their adhesion to endothelial cells while in macrophages and lymphocytes increased IL-10 production, inducing anti-inflammatory effects [19]. In this same line, it has been demonstrated that expression of A2b is induced when lung cells are exposed to pro-inflammatory stimuli. This mechanism would produce a damping of inflammation and lung protection [62,63]. Moreover, the use of the specific antagonist for A2b, MRS1754 increased the inflammatory effects induced by LPS [62,63]. Similarly, pharmacological treatment with the agonist BAY 60-6583 reduces lung inflammation and edema caused by acute lung injury induced by LPS in mice [62,64] and also decrease TNF-α levels [63]. For these anti-inflammatory effects, it has been suggested that BAY 60-6583 could be used as an add-on therapy in some glucocorticoid-resistant lung diseases [65].

On the contrary, several studies have demonstrated pro-inflammatory effects of A2b. The activation of this receptor in mast cells produces degranulation and secretion of pro-inflammatory mediators such as IL-1β, IL-13, IL-3, IL-8 and IL-4 [19], leading to bronchoconstriction [50]. The use of a specific A2b antagonist, CVT-6883, attenuated inflammation and reduced bronchoconstriction [19,50]. Moreover, CVT-6883 treatment in a mice model based on multi-walled carbon nanotube-induced pulmonary fibrosis, reduces the levels of pro-fibrotic mediators [66]. In addition, treatment with another antagonist, GS6201, decreased the hypertension associated with interstitial lung disease [19]. Furthermore, the administration of the A2b antagonist ATL802 in lung injury caused by ischemia-reperfusion, attenuated inflammation and pro-inflammatory cytokines release, improving lung function, decreasing resistance, blood pressure, and pulmonary vascular permeability [67].

#### 3.1.4. A3 

The activation of A3 is related with pro-inflammatory and anti-inflammatory effects depending on the cell type [7]. In the airways of asthmatics, A3 is found to be mainly expressed in eosinophils [50]. The activation of A3 induced the inhibition of eosinophil degranulation and chemotaxis. In fact, it has been hypothesized that the use of A3 agonists may be a useful therapeutic approach for eosinophil-dependent allergic diseases [5]. On the contrary, the activation of A3 in both rodent and human LAD2 mast cells enhanced degranulation induced by FcεRI activation [5,7,48]. Interestingly, low adenosine concentrations enhanced FcεRI-mediated degranulation in human lung, but not in skin mast cells. A3 overexpression in human lung mast cell may account for this differential effect [31]. In fact, the activation of A3 by a specific agonist, CI-IB-MECA, increased the expression of genes involved in vascular remodeling, such as IL-6, IL-8, and VEGF, and pro-fibrotic genes, such as osteopontin and amphiregulin, which leads to a worsening of asthma [30].

## 4. Discussion

Adenosine is a nucleoside that produces both pro-inflammatory and anti-inflammatory effects depending on the model used. Adenosine acts through the binding to specific receptors that are widely distributed throughout the human body [7,68]. In fact, adenosine produces opposite effects depending on its concentration, cell type, organ and species [48].

These different effects could be partially explained by the diverse pattern and level of expression of AR in each cell type [31,32,33,55]. However, other factors such as the desensitization susceptibility, capacity of receptor up-regulation in response a pathological condition, as well as the adenosine concentration present in the cell environment may also account for these differences. For these reasons, ARs continuous activation under pathological conditions may results in different scenarios that may go from anti-inflammatory to pro-inflammatory situations. On the other hand, and related to the AR up-regulation, it has been demonstrated that inflammatory environment induces A2a and A2b expression [57,62,63], as a part of a compensatory mechanism. Finally, it has been demonstrated that low (<1 µM) and high (1000 µM) adenosine concentrations had opposite effects in the same cell type; histamine release induced by FcεRI in human lung mast cell is either enhanced or inhibited depending on the adenosine concentration [49].

ATP and ADP are precursors of adenosine and purinergic members involved in allergic diseases and exert their effects through binding to P2 receptors; ionotropic P2X and metabotropic P2Y receptors. ATP can bind to both P2X and P2Y, although ADP can only bind to P2Y. In mast cells, both P2Y and P2X have been shown to increase degranulation [29,69,70] suggesting that they may contribute to the final effect of adenosine in allergic disease and that may also be study targets in the allergy field.

Considering the evidence summarized in this review that highlights the complexity of adenosine effects in mast cells, it seems difficult to elucidate the precise mechanism of action of adenosine in the different inflammatory and allergic diseases. However, the anti-inflammatory effects of the A2a seem consistent through the different models, suggesting that A2a agonists may become effective therapies in the future. In fact, different A2a agonists have been used as therapies for lung inflammatory disease, although unwanted cardiovascular effects, suggest that a more selective organ targeting is required [71,72].

## 5. Conclusions

In summary, the adenosine signaling pathway has an impact on allergic and inflammatory diseases. Therefore, the development of specific agonist and antagonist targeting a particular receptor in the target organ, may be the key for more efficient therapies.

## References

[1] Sachdeva S., Gupta M. (2013). Adenosine and its receptors as therapeutic targets: An overview. Saudi Pharm. J..

[2] Le T.-T.T., Berg N.K., Harting M.T., Li X., Eltzschig H.K., Yuan X. (2019). Purinergic Signaling in Pulmonary Inflammation. Front. Immunol..

[3] Sun Y., Huang P. (2016). Adenosine A2B Receptor: From Cell Biology to Human Diseases. Front. Chem..

[4] Borea P.A., Gessi S., Merighi S., Varani K. (2016). Adenosine as a Multi-Signalling Guardian Angel in Human Diseases: When, Where and How Does it Exert its Protective Effects?. Trends Pharmacol. Sci..

[5] Borea P.A., Varani K., Vincenzi F., Baraldi P.G., Tabrizi M.A., Merighi S., Gessi S. (2015). The A3Adenosine Receptor: History and Perspectives. Pharmacol. Rev..

[6] Antonioli L., Fornai M., Blandizzi C., Pacher P., Haskó G. (2019). Adenosine signaling and the immune system: When a lot could be too much. Immunol. Lett..

[7] Borea P.A., Gessi S., Merighi S., Vincenzi F., Varani K. (2018). Pharmacology of Adenosine Receptors: The State of the Art. Physiol. Rev..

[8] Giuliani A.L., Sarti A.C., Di Virgilio F. (2019). Extracellular nucleotides and nucleosides as signalling molecules. Immunol. Lett..

[9] Van Linden A., Eltzschig H.K. (2007). Role of pulmonary adenosine during hypoxia: Extracellular generation, signaling and metabolism by surface adenosine deaminase/CD26. Expert Opin. Biol. Ther..

[10] Haskó G., Antonioli L., Cronstein B.N. (2018). Adenosine metabolism, immunity and joint health. Biochem. Pharmacol..

[11] Zhou Y., Murthy J.N., Zeng D., Belardinelli L., Blackburn M.R. (2010). Alterations in Adenosine Metabolism and Signaling in Patients with Chronic Obstructive Pulmonary Disease and Idiopathic Pulmonary Fibrosis. PLoS ONE.

[12] Idzko M., Ayata C.K., Müller T., Dürk T., Grimm M., Baudiß K., Vieira R.P., Cicko S., Boehlke C., Zech A. (2013). Attenuated allergic airway inflammation inCd39null mice. Allergy.

[13] Moreno E., Canet J., Gracia E., Lluis C., Mallol J., Canela E.I., Cortés A., Casadó V. (2018). Molecular Evidence of Adenosine Deaminase Linking Adenosine A2A Receptor and CD26 Proteins. Front. Pharmacol..

[14] Boison D. (2018). Regulation of Extracellular Adenosine. Nicotinic Recept..

[15] Zhong H., Chunn J.L., Volmer J.B., Fozard J.R., Blackburn M.R. (2001). Adenosine-mediated mast cell degranulation in adenosine deaminase-deficient mice. J. Pharmacol. Exp. Ther..

[16] Jin X., Shepherd R.K., Duling B.R., Lindén J. (1997). Inosine binds to A3 adenosine receptors and stimulates mast cell degranulation. J. Clin. Investig..

[17] Yu M., Cui F.-X., Jia H.-M., Zhou C., Yang Y., Zhang H.-W., Ding G., Zou Z.-M. (2016). Aberrant purine metabolism in allergic asthma revealed by plasma metabolomics. J. Pharm. Biomed. Anal..

[18] Young J.D. (2016). The SLC28 (CNT) and SLC29 (ENT) nucleoside transporter families: A 30-year collaborative odyssey. Biochem. Soc. Trans..

[19] Effendi W.I., Nagano T., Kobayashi K., Nishimura Y. (2020). Focusing on Adenosine Receptors as a Potential Targeted Therapy in Human Diseases. Cells.

[20] Sheth S., Brito R., Mukherjea D., Rybak L.P., Ramkumar V. (2014). Adenosine Receptors: Expression, Function and Regulation. Int. J. Mol. Sci..

[21] Merighi S., Gessi S., Borea P.A. (2018). Adenosine Receptors: Structure, Distribution, and Signal Transduction. Nicotinic Recept..

[22] Alnouri M.W., Jepards S., Casari A., Schiedel A.C., Hinz S., Müller C.E. (2015). Selectivity is species-dependent: Characterization of standard agonists and antagonists at human, rat, and mouse adenosine receptors. Purinergic Signal..

[23] Peleli M., Fredholm B.B., Sobrevia L., Carlström M. (2017). Pharmacological targeting of adenosine receptor signaling. Mol. Asp. Med..

[24] Chen J.-F., Eltzschig H.K., Fredholm B.B. (2013). Adenosine receptors as drug targets—What are the challenges?. Nat. Rev. Drug Discov..

[25] Gao Z.-G., Tosh D.K., Jain S., Yu J., Suresh R.R., Jacobson K.A. (2018). A1 Adenosine Receptor Agonists, Antagonists, and Allosteric Modulators. Nicotinic Recept..

[26] Ciancetta A., Jacobson K.A. (2017). Structural Probing and Molecular Modeling of the A3 Adenosine Receptor: A Focus on Agonist Binding. Molecules.

[27] Méndez-Enríquez E., Hallgren J. (2019). Mast Cells and Their Progenitors in Allergic Asthma. Front. Immunol..

[28] Matsuo Y., Yanase Y., Irifuku R., Ishii K., Kawaguchi T., Takahagi S., Hide I., Hide M. (2018). The role of adenosine for IgE receptor-dependent degranulation of human peripheral basophils and skin mast cells. Allergol. Int..

[29] Gao Z.-G., Jacobson K.A. (2017). Purinergic Signaling in Mast Cell Degranulation and Asthma. Front. Pharmacol..

[30] Rudich N., Dekel O., Sagi-Eisenberg R. (2015). Down-regulation of the A3 adenosine receptor in human mast cells upregulates mediators of angiogenesis and remodeling. Mol. Immunol..

[31] Gomez G., Zhao W., Schwartz L.B. (2011). Disparity in FcεRI-Induced Degranulation of Primary Human Lung and Skin Mast Cells Exposed to Adenosine. J. Clin. Immunol..

[32] Muñoz-Cano R., Pascal M., Bartra J., Picado C., Valero A., Kim D.-K., Brooks S., Ombrello M., Metcalfe D.D., Rivera J. (2016). Distinct transcriptome profiles differentiate nonsteroidal anti-inflammatory drug–dependent from nonsteroidal anti-inflammatory drug–independent food-induced anaphylaxis. J. Allergy Clin. Immunol..

[33] Kim S.-H., Nam E.-J., Kim Y.-K., Ye Y.-M., Park H.-S. (2010). Functional variability of the adenosine A3 receptor (ADORA3) gene polymorphism in aspirin-induced urticaria. Br. J. Dermatol..

[34] Kim S.-H., Kim Y.-K., Park H.-W., Kim S.-H., Ye Y.-M., Min K.-U., Park H.-S. (2009). Adenosine deaminase and adenosine receptor polymorphisms in aspirin-intolerant asthma. Respir. Med..

[35] Liu Y., Saccucci P., Qi H., Wu H.C., Zhao F., Dai Y., Bottini N., Gloria-Bottini F. (2006). ADA Polymorphisms and Asthma: A Study in the Chinese Han Population. J. Asthma.

[36] Marone G., Findlay S.R., Lichtenstein L.M. (1979). Adenosine receptor on human basophils: Modulation of histamine release. J. Immunol..

[37] Jacobson K.A., Gao Z.-G. (2006). Adenosine receptors as therapeutic targets. Nat. Rev. Drug Discov..

[38] Guerrero Á. (2018). A2A Adenosine Receptor Agonists and their Potential Therapeutic Applications. An Update. Curr. Med. Chem..

[39] Merighi S., Bencivenni S., Vincenzi F., Varani K., Borea P.A., Gessi S. (2017). A 2B adenosine receptors stimulate IL-6 production in primary murine microglia through p38 MAPK kinase pathway. Pharmacol. Res..

[40] Jacobson K.A., Merighi S., Varani K., Borea P.A., Baraldi S., Tabrizi M.A., Romagnoli R., Baraldi P.G., Ciancetta A., Tosh D.K. (2018). A3Adenosine Receptors as Modulators of Inflammation: From Medicinal Chemistry to Therapy. Med. Res. Rev..

[41] Klaasse E.C., Ijzerman A.P., De Grip W.J., Beukers M.W. (2007). Internalization and desensitization of adenosine receptors. Purinergic Signal..

[42] Mundell S., Kelly E. (2011). Adenosine receptor desensitization and trafficking. Biochim. Biophys. Acta BBA Biomembr..

[43] Stoddart L.A., Vernall A.J., Briddon S.J., Kellam B., Hill S.J. (2015). Direct visualisation of internalization of the adenosine A3 receptor and localization with arrestin3 using a fluorescent agonist. Neuropharmacology.

[44] Soave M., Kellam B., Woolard J., Briddon S.J., Hill S.J. (2020). NanoBiT Complementation to Monitor Agonist-Induced Adenosine A1 Receptor Internalization. SLAS Discov. Adv. Life Sci. R&D.

[45] Cox C.A., Boudewijn I.M., Vroegop S.J., Schokker S., Lexmond A.J., Frijlink H.W., Hagedoorn P., Vonk J.M., Farenhorst M.P., Hacken N.H.T.T. (2019). Associations of AMP and adenosine induced dyspnea sensation to large and small airways dysfunction in asthma. BMC Pulm. Med..

[46] Wilson C.N. (2008). Adenosine receptors and asthma in humans. Br. J. Pharmacol..

[47] Mao M., Liu H., Yan S., Yuan Y., Liu R., Wu Y., Peng C., Li J., Chen X. (2021). Plasma adenosine is linked to disease activity and response to treatment in patients with chronic spontaneous urticaria. Allergy.

[48] Leung C.T., Li A., Banerjee J., Gao Z.-G., Kambayashi T., Jacobson K.A., Civan M.M. (2014). The role of activated adenosine receptors in degranulation of human LAD2 mast cells. Purinergic Signal..

[49] Schulman E.S., Glaum M.C., Post T., Wang Y., Raible D.G., Mohanty J., Butterfield J.H., Pelleg A. (1999). ATP Modulates Anti-IgE–Induced Release of Histamine from Human Lung Mast Cells. Am. J. Respir. Cell Mol. Biol..

[50] Caruso M., Alamo A., Crisafulli E., Raciti C., Fisichella A., Polosa R. (2013). Adenosine signaling pathways as potential therapeutic targets in respiratory disease. Expert Opin. Ther. Targets.

[51] Fernandez L.G., Sharma A.K., LaPar D.J., Kron I.L., Laubach V.E. (2013). Adenosine A1 receptor activation attenuates lung ischemia–reperfusion injury. J. Thorac. Cardiovasc. Surg..

[52] Patel M., Narke D., Kurade M., Frey K.M., Rajalingam S., Siddiquee A., Mustafa S.J., Ledent C., Ponnoth D.S. (2020). Limonene-induced activation of A2A adenosine receptors reduces airway inflammation and reactivity in a mouse model of asthma. Purinergic Signal..

[53] Ohta A., Sitkovsky M. (2001). Role of G-protein-coupled adenosine receptors in downregulation of inflammation and protection from tissue damage. Nat. Cell Biol..

[54] Kong X., Zuo Y., Huang Y., Ge J. (2019). Adenosine A2a receptor agonist CGS21680 treatment attenuates cardiopulmonary bypass-associated inflammatory lung injury in juvenile rats. Mol. Med. Rep..

[55] Alfaro T.M., Rodrigues D.I., Tomé Â.R., Cunha R.A., Cordeiro C.R. (2017). Adenosine A 2A receptors are up-regulated and control the activation of human alveolar macrophages. Pulm. Pharmacol. Ther..

[56] Pei H., Linden J. (2016). Adenosine influences myeloid cells to inhibit aeroallergen sensitization. Am. J. Physiol. Cell. Mol. Physiol..

[57] Yuryeva K., Saltykova I., Ogorodova L., Kirillova N., Kulikov E., Korotkaya E., Iakovleva Y., Feoktistov I., Sazonov A., Ryzhov S. (2015). Expression of adenosine receptors in monocytes from patients with bronchial asthma. Biochem. Biophys. Res. Commun..

[58] Duffy S.M., Cruse G., Brightling C.E., Bradding P. (2007). Adenosine closes the K+ channel KCa3.1 in human lung mast cells and inhibits their migrationvia the adenosine A2A receptor. Eur. J. Immunol..

[59] Suzuki H., Takei M., Nakahata T., Fukamachi H. (1998). Inhibitory Effect of Adenosine on Degranulation of Human Cultured Mast Cells upon Cross-Linking of FceRI. Biochem. Biophys. Res. Commun..

[60] Arizmendi N., Kulka M. (2018). Adenosine activates Gαs proteins and inhibits C3a-induced activation of human mast cells. Biochem. Pharmacol..

[61] Hua X., Kovarova M., Chason K.D., Nguyen M., Koller B.H., Tilley S.L. (2007). Enhanced mast cell activation in mice deficient in the A2b adenosine receptor. J. Exp. Med..

[62] Schingnitz U., Hartmann K., MacManus C.F., Eckle T., Zug S., Colgan S.P., Eltzschig H.K. (2010). Signaling through the A2B Adenosine Receptor Dampens Endotoxin-Induced Acute Lung Injury. J. Immunol..

[63] Cohen H.B., Ward A., Hamidzadeh K., Ravid K., Mosser D.M. (2015). IFN-γ Prevents Adenosine Receptor (A2bR) Upregulation to Sustain the Macrophage Activation Response. J. Immunol..

[64] Hoegl S., Brodsky K.S., Blackburn M.R., Karmouty-Quintana H., Zwissler B., Eltzschig H.K. (2015). Alveolar Epithelial A2B Adenosine Receptors in Pulmonary Protection during Acute Lung Injury. J. Immunol..

[65] Greer S., Page C.W., Joshi T., Yan N., Newton R., Giembycz M.A. (2013). Concurrent Agonism of Adenosine A2B and Glucocorticoid Receptors in Human Airway Epithelial Cells Cooperatively Induces Genes with Anti-Inflammatory Potential: A Novel Approach to Treat Chronic Obstructive Pulmonary Disease. J. Pharmacol. Exp. Ther..

[66] Liu B., Bing Q., Li S., Han B., Lu J., Baiyun R., Zhang X., Lv Y., Wu H., Zhang Z. (2019). Role of A2B adenosine receptor-dependent adenosine signaling in multi-walled carbon nanotube-triggered lung fibrosis in mice. J. Nanobiotechnol..

[67] Huerter M.E., Sharma A.K., Zhao Y., Charles E.J., Kron I.L., Laubach V.E. (2016). Attenuation of Pulmonary Ischemia-Reperfusion Injury by Adenosine A 2B Receptor Antagonism. Ann. Thorac. Surg..

[68] Jamwal S., Mittal A., Kumar P., Alhayani D.M., Al-Aboudi A. (2019). Therapeutic Potential of Agonists and Antagonists of A1, A2a, A2b and A3 Adenosine Receptors. Curr. Pharm. Des..

[69] Yoshida K., Ito M., Hoshino Y., Matsuoka I. (2017). Effects of dexamethasone on purinergic signaling in murine mast cells: Selective suppression of P2X7 receptor expression. Biochem. Biophys. Res. Commun..

[70] Yoshida K., Ito M.-A., Sato N., Obayashi K., Yamamoto K., Koizumi S., Tanaka S., Furuta K., Matsuoka I. (2020). Extracellular ATP Augments Antigen-Induced Murine Mast Cell Degranulation and Allergic Responses via P2X4 Receptor Activation. J. Immunol..

[71] Müller C.E., Jacobson K.A. (2011). Recent developments in adenosine receptor ligands and their potential as novel drugs. Biochim. Biophys. Acta BBA Biomembr..

[72] Åstrand A.B.M., Bergström E.L., Zhang H., Börjesson L., Söderdahl T., Wingren C., Jansson A., Smailagic A., Johansson C., Bladh H. (2015). The discovery of a selective and potent A 2a agonist with extended lung retention. Pharmacol. Res. Perspect..

